# Health and Hunger: Disease, Energy Needs, and the Indian Calorie Consumption Puzzle*

**DOI:** 10.1111/ecoj.12417

**Published:** 2017-04-27

**Authors:** Josephine Duh, Dean Spears

**Affiliations:** University of Texas at Austin, Indian Statistical Institute – Delhi Centre, and r.i.c.e

## Abstract

India’s experience presents a puzzle at odds with a basic fact of household economics: amidst unprecedented economic growth, average *per capita* daily calorie consumption has declined in recent decades. Does an improving disease environment explain the calorie decline? A diminished burden of infectious disease could lower energy needs by increasing absorption and effective use of calories. We document a robust effect of disease exposure - measured as infant mortality and as poor sanitation - on calorie consumption. Similar effects are found using multiple datasets and empirical strategies. Disease can account for an important fraction (one-fifth or more) of India’s calorie decline.

Food consumption has long been central to household economics and the measurement of well-being. At least since the nineteenth century, it has been documented that, particularly in developing countries, richer households consume more food on average than poorer households^1^. However, recent trends in India challenge our understanding of the basic facts of household economics and present a major puzzle. With rapid economic growth, the average Indian household has become richer over time. However, calorie consumption has been declining over recent decades (Deaton and Drèze, 2009). From 1987-8 to 2004-5, the average daily *per capita* calorie consumption decreased by about 130 calories, or 6%, and calories from cereals^2^ fell by about 200 calories, or 14%.

What factors explain India’s decline in calorie consumption amidst economic growth? This article investigates one novel hypothesis: calorie consumption has fallen in part because slow improvements in India’s disease environment have lowered energy intake needs. India has exceptionally poor sanitation: 60% of people worldwide who defecate in the open without using a toilet or latrine live in India.^3^ Partially in consequence, India’s infant mortality rate is higher than that of other countries with similar levels of national income *per capita*. Reductions in this considerable burden of disease may have allowed Indian consumers to retain, absorb and use more of the food that they eat. Because many developing countries, including India, continue to suffer from much preventable disease, it is important to understand the economic impact of disease on average calorie needs.

This article makes two contributions to the literature. To our knowledge, we are the first to estimate an importantly large, but plausibly sized, effect of the disease environment on average calorie consumption. Households exposed to a greater burden of infectious disease eat more. In addition, we present empirical evidence that improvements in the disease environment can account for a large fraction - possibly one-fifth or more - of the recent calorie consumption decline in India. Although none of our estimates suggest that disease can account for the entire calorie decline, our results advance an emerging literature highlighting the key role of disease externalities for nutrition (Smith *et al*., 2013; Spears, 2013).

Employing two empirical strategies and three separate datasets with complementary advantages, we estimate comparably sized effects of the disease environment on calorie consumption. Indian districts that experienced larger declines in infant mortality from the mid-1980s to mid-2000s also saw larger decreases in average *per capita* calorie consumption. Similarly, the average household living in an area with worse sanitation and higher infant mortality consumed more calories than otherwise comparable households living in places with lower rates of open defecation and infant mortality. Effects are pronounced in areas where more children suffer from diarrhoea; no similar effect is seen of fever or cough.

In the absence of an effect of the disease environment on nutritional needs, this result would appear paradoxical: generally, poorer people suffer worse disease and eat less. Alternative causal explanations are challenged to account for the fact that eating more - which is typically associated with socio-economic advantage - robustly coincides with exposure to disease externalities - which are typically associated with socioeconomic disadvantage.

## 1 Motivation: Three Puzzles of Nutrition and Consumption

From the 1980s to the 2000s, the average daily calorie consumption in India decreased by an economically important amount. As Deaton and Drèze (2009, p. 42), summarise, ‘this decline has occurred across the distribution of real *per capita* expenditure, in spite of increases in real income and no long-term increase in the relative price of food’. Perhaps most puzzlingly, this decline occurred during an exceptional period of rapid economic growth in India: real GDP *per capita* grew at 4% per year from 1983 to 2005.^4^ Among a wide set of candidate explanations that they consider, Deaton and Dreeze (2009) hypothesise that ‘calorie requirements have declined due to lower levels of physical activity or improvements in the health environment’. Eli and Li (2013) carefully consider the first suggestion and find that changing work requirements and physical activity are unlikely to account for more than one-third of India’s calorie decline. Since much of the calorie decline remains to be explained, we consider the effects of health: if an improved disease environment allows for better absorption and efficient use of caloric intake, how much of the decline could disease explain?

Although we do not directly address it in this article, a related nutritional puzzle in India concerns anthropometric outcomes. People in India are exceptionally short in international comparisons, especially given their relatively high average incomes among developing countries (Deaton, 2007). It is particularly puzzling that people in India are shorter, on average, than people in sub-Saharan Africa who are poorer, on average; this fact is sometimes called the ‘Asian Enigma’ (Ramalingaswami et *al*., 1996) and has received much attention from economists (Tarozzi, 2008; Jayachandran and Pande, 2013).^5^ In a related finding that motivates our analysis, Spears (2013) demonstrates that children in populations exposed to more open defecation are shorter, on average; exceptionally poor sanitation in India can statistically account for India’s deficit relative to Africa in child height. Economic historians have documented a large association between population height and the disease environment, as reflected in mortality rates (Bozzoli *et al*., 2009). Hatton (2013, p. 1), studying the historical increase in European height, concludes that ‘the most important proximate source of increasing height was the improving disease environment as reflected by the fall in infant mortality’.

A third motivating nutritional puzzle is the limited success of interventions that aim to improve nutritional outcomes (such as child height) through direct provision of food or nutrients. Numerous field experiments have documented limited success of nutritional supplementation. Describing these as ‘nutrition-specific interventions’, Bhutta *et al*. (2013) estimate that if 10 core nutrition-specific interventions were scaled- up to 90%, child stunting would fall by about 20%. If disease interacts with nutrient intake (Menon *et al*., 2013) such that a reduced burden of disease allows children’s bodies to absorb and make more efficient use of nutrients from the intervention, then ‘nutrition-sensitive interventions’ - such as improving the disease environment - may help to bridge the gap.

This article builds on an important literature in economic history on nutrition and the demand for calories. Much of this literature studies Europe in the eighteenth to twentieth centuries, where health consequences of sanitary environments have received considerable attention (Preston and van de Walle, 1978; Fogel, 1997). In a puzzle sharing similarities to the Indian puzzle that we study, Clark *et al.* (1995) show that food material supplies decreased in Britain from 1770 to 1850, despite growth in income. They allude to the possibility of changes in food demand related to disease but do not test for it directly. Given that the first treated water supply in London was not until 1829 and that the 1855 Metropolitan Water Act came after the sample period, disease may not be the primary driving force behind the demand shift in their study. Logan (2009) finds that calorie expenditure elasticities among 19th-century industrial workers were much greater than the elasticities among similarly poor or poorer people in developing countries today, suggesting that these historical workers were hungrier. One reason why the industrial workers were hungrier may be that, holding standard of living constant, sanitation and the disease environment was considerably worse historically, before widespread acceptance of the germ theory of disease.^6^ Deaton (2006, p. 111), reviewing Fogel and reflecting on this historical and anthropometric literature, expresses concern that ‘the synergism between economic growth and the growth of the size and the durability of the human body can turn into an overemphasis on links between economic growth and health and an underemphasis on the role of disease and its prevention’. If so, then this article’s estimate of effects of high disease burdens on calorie demand is an important step towards understanding a long- discussed, but under-quantified mechanism.

### 1.1 Links Between the Disease Environment and Nutrition

Could a greater burden of disease increase calorie needs - either because nutrients eaten are not absorbed due to diarrhoea, parasites, or intestinal dysfunction, or because the body uses energy fighting disease (Stephensen, 1999)? As Deaton (2007) explains, anthropometric outcomes reflect ‘net nutrition’, meaning nutrient intake net of losses to disease. If disease does increase nutritional requirements, then people in India would face an exceptional risk: more than half of households in India defecate in the open without using a toilet or latrine (WHO and Unicef, 2012 and open defecation is just one of many sources of infectious disease.

Although no prior paper in economics has sought to estimate an effect of the disease environment on calorie consumption, a substantial econometric literature documents effects of sanitation and water on health. Cutler and Miller (2005) document a large effect of water filtration and chlorination on mortality in major US cities in the early 20th century. Similarly, Watson (2006) studied heterogeneous timing of public health investments - including sewer connections and septic tanks at US Indian reservations. Watson found that a 10 percentage point increase in the fraction of homes receiving improved sanitation reduced infant mortality by 2.5% among Native Americans. Galiani *et al*. (2005) show that privatisation of water supply in Argentina reduced child mortality by 8%.

Other papers in economics trace effects of sanitation onto nutritional outcomes and their long-term consequences for human capital (Lawson and Spears, 2016). Bleakley (2007) documented that eliminating hookworm in the American South led to an increase in literacy and average incomes; worms and other parasites are a key mechanism by which disease could increase food intake needs. Baird *et al*. (2011), following up on Miguel and Kremer (2004) deworming experiment in Kenyan schools, found that children who received the deworming treatment grew up to be adults who work more hours. Lastly, Spears (2012*a)* and Spears and Lamba (2016) studied a government sanitation programme in rural India. Exploiting heterogeneity in implementation throughout rural India, they find that the where the programme was active, it reduced infant mortality, increased children’s height and subsequently increased academic test scores, on average.

A growing biomedical literature on links between sanitation, disease and nutrition is consistent with the possibility of an effect of the disease environment on calorie demand, due to nutrient absorption and use. Research on the interaction between infection and nutrition has built upon early insights of Scrimshaw *et al.* (1968).^7^ This literature on nutritional consequences of infectious disease has traditionally concentrated on diarrhoea (Guerrant et *al.,* 1992; Checkley *et al*., 2008). However, recent research has concentrated on the possible importance of *environmental enteric dysfunction* (EED), an inflammatory response of the intestines to chronic infection, resulting in reduced nutrient absorption (Humphrey, 2009; Mondal *et al*., 2011). Recent epidemiological studies have exploited novel methods to measure markers of EED, and have found strong associations among environmental sanitation, EED and nutritional outcomes (Kosek *et al*., 2013; Lin *et al*., 2013). Although none of these studies have measured an economic response to these conditions, both diarrhoea and malabsorption due to EED as well as energy demands of fighting disease could be consistent with increased demand for calories where the disease burden is greater.

### 1.2 Outline

We conduct three complementary empirical analyses to estimate effects of the disease environment on calorie consumption. In our empirical analyses, we use two measures of disease externalities: infant mortality and open defecation. Infant mortality rates have long been used as a measure of the disease environment by economic historians, and have been shown to correlate with anthropometric nutritional outcomes (Bozzoli *et al*., 2009; Hatton, 2013), although to our knowledge IMR has never previously been linked directly with increase calorie consumption. Open defecation is an important source of disease in India and can explain variation in child height internationally and within India (Spears, 2013). Finding similar results with both explanatory variables contributes to our interpretation of our estimates as reflective of an effect of the disease environment.

First, in Section 2, we combine two surveys of food consumption in India, one from 1987/88 and one from 2004/05, to create a district-level panel from repeated crosssections. Having merged consumption data with infant mortality rates from the Indian Census, we employ a difference-in-differences identification strategy: districts that experienced sharper declines in infant mortality rates over these two decades also saw greater decreases in calorie consumption, conditional on overall household expenditure. Second, in Section 3, we study a nationally representative cross-sectional survey that uniquely combines data on food consumption, mortality, sanitation and anthropometric outcomes. We show that households living in rural villages or urban blocks with higher rates of infant mortality (or open defecation) consume more calories, on average; we also confirm that adult women living in areas with more disease have lower body mass, even after accounting for differences in household expenditure and calorie consumption. Finally, in Section 4, we use a unique survey dataset from 1983 which combines data on consumption and local sanitation with demography- specific employment information within households; this allows us to replicate our main result while verifying that variation in energy requirements for work are unlikely to drive our results. Section 5 quantitatively compares the estimates from these three approaches and applies decomposition methods in the spirit of Blinder-Oaxaca to estimate the fraction of the Indian calorie decline that can be explained by improvements in the disease environment.

## 2 Evidence from Changes Over Time Within Districts

Did average *per capita* calorie consumption fall more steeply in districts where infant mortality rates more sharply declined between 1987-8 and 2004-5? In this Section, we answer this question by combining two cross-sectional rounds of India’s National Sample Survey (Rounds 43 and 61) in order to use a panel-based identification strategy (Deaton, 1985). We focus on these two survey rounds because the data allow us to identify the districts in which households live; we then match districts across years and merge the consumption data with infant mortality records from the Indian census. By focusing on within-district changes in calorie consumption and infant mortality, we are able to rule out confounding time-invariant factors, such as climate or average genetic potential body size of the local population. We first describe the data and our empirical strategy in subsection 2.1, and then, in subsection 2.2, we present the regression results.

### 2.1 Empirical Strategy

Our outcome variable is daily *per capita* calorie consumption from the National Sample Survey (NSS). The NSS is a nationally representative household-based survey conducted by the National Sample Survey Office (NSSO) in the Ministry of Statistics and Programme Implementation. The NSSO annually fields the Consumer Expenditure Survey (CES) and Employment-Unemployment Survey (EUS) using a two-stage sample design covering all Indian states.^8^

We follow Deaton and Drèze (2009) in calculating calorie consumption from households’ 30-day recall of food expenditures in the CES. Households are asked to report quantities of more than 200 items consumed from home production, market purchase and free collection or gifts, and households are probed for total spending on each item purchased from the market. Using conversion factors from Nutritive Value of Indian Foods by Gopalan *et al*. (1989) provided by the NSSO survey reports, we are able to translate quantities into calories; we divide by the household size to obtain average calories consumed per person.

Our explanatory variable that measures the disease environment is the district infant mortality rate (IMR). IMR is defined as the number of deaths among babies less than 12 months old per 1,000 live births. Because infants are highly sensitive to respiratory infections and intestinal diseases, the literature has frequently used IMR to proxy for the prevailing disease environment (Bozzoli *et al*., 2009; Hatton, 2013). Since the NSS does not record information on infant mortality, we merge NSS data with district-level IMR from the Indian Census.^9^

Summary statistics of NSS Rounds 43 and 61 are presented in columns (1) and (2) of Table 1. On average, daily calorie consumption per person decreased by 126 calories (or 5.8% of average consumption level in 1987/88) between 1987/88 and 2004/05. The decline was mainly driven by a drop of 213 cereal calories (or 13.8%) per person.

**Table 1:** Sample Statistics for National Sample Survey Rounds 38/43/61 and India Human Development Survey 2005

	NSS Round 43 (1987-8)	NSS Round 61 (2004-5)	IHDS(2005)	NSS Round 38 (1983)
*Per capita* consumption of cereal calories	1,550	1,337	1,254	1,564
*Per capita* consumption of all calories	2,172	2,046	1,787	2,140
District IMR (per 1,000 live births)	89	58	45	-
Percentage of households without toilets in district (NSS 43 & 61) or PSU (IHDS & NSS 38)	80.7	60.9	58.4	79.2
Urban	0.225	0.248	0.290	0.213
MPCE (in 1987-8 Rupees)	174	214	799	116
Food expenditure as share of HH budget	0.723	0.666	0.555	0.735
Self-employed in agriculture (NSS) or Cultivation (IHDS)	0.331	0.297	0.246	0.362
Agricultural labour	0.317	0.285	0.155	0.217
Has a TV	0.024	0.391	0.486	-
Has a motorised vehicle	0.042	0.151	0.161	-
No adult female is literate	0.606	0.415	0.483	-
No adult male is literate	0.308	0.197	0.236	-
N (households)	115,114	113,123	38,227	108,330

*Notes.* Samples exclude households in the top and bottom 1% of cereal calories distribution in rural and urban sectors. MPCE stands for monthly *per capita* expenditures. For Round 38, sample only includes households that matched in both in the Consumer Expenditure Survey and Employment-Unemployment Survey. MPCE for NSS Round 38 (1983) is reported in 1983 Rupees.

As calorie consumption was falling, the disease environment and socio-economic conditions were improving. In the late 1980s, the average Indian household lived in a district where IMR was 89 deaths per 1,000 live births; by the mid-2000s, IMR decreased to 58 deaths per 1,000. Over the same period, latrine coverage increased, which contributed to the reduction of disease (Spears, 2012a). Real^10^ monthly *per capita* expenditures rose by 23%, and reported ownership of a TV increased by 14-fold. The fraction of illiterate women in the population was cut by nearly one-third and almost one-half for men.

To estimate the effect of the disease environment on calorie consumption, we use a fixed effects model that also allows us to control for household wealth, primary source of income and education:

(1) $$calories_{idt}=\beta_{0}+\beta_{1}IMR_{dt}+\beta_{2}\ln(MPCE{)}_{idt}+X_{idt}\theta +\gamma_{d}+\delta_{t}+\varepsilon_{idt},$$

where *i* indexes households, *d* districts, and *t* years, here survey rounds. The outcome variable (*calories_idt_*) is *per capita* consumption of total calories or cereal calories and the key explanatory variable is the district’s infant mortality rate during the survey year (*IMR_dt_*). To make use of the available information on households that may also correlate with calorie consumption, we conduct the analysis at the household level, and since IMR varies by district, we cluster standard errors at the district level.^11^

We add covariates (*X_idt_*) in stages to demonstrate the stability of our result. We include an indicator for urban residence, the household’s monthly *per capita* expenditures (MPCE) in logs, caste and religion of the household head, literacy of adult male or female, and 34 categorical variables of the household’s primary occupation. We include district (y_d_) and time (d_t_) fixed effects. Note that, with district fixed effects, the coefficients are estimated based on changes within districts over time. Finally, we replicate all regressions with and without detailed semi-parametric controls for the count of household members of each sex in a set of age ranges, to verify that no demographic properties of households are responsible for our results.^12^

Our preferred results focus on calories from cereals rather than calories from all foods because we are interested in the effect of the disease environment on calorie needs: in most cases, cereals are the main and cheapest source of energy (Jensen and Miller, 2010). Nevertheless, as a robustness check, we also show results using calories from all food groups as the outcome variable.

### 2.2 Effect of the Disease Environment on Calories Consumption

**Fig. 1:**
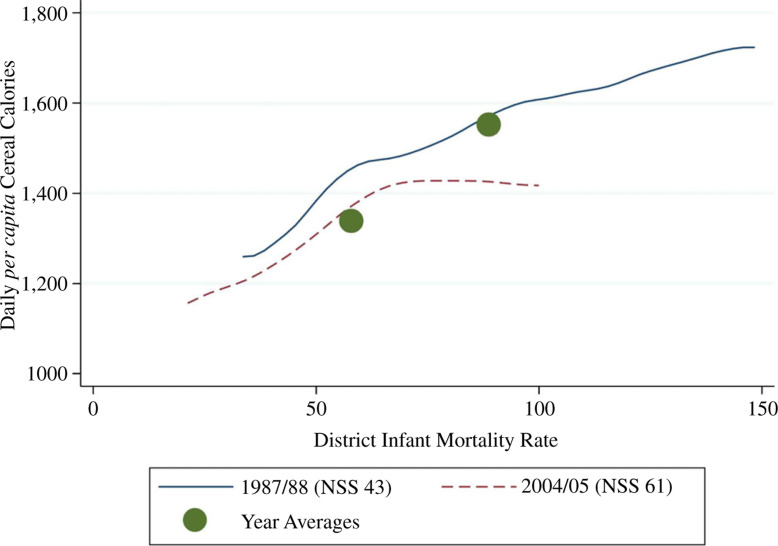
Cereal Calorie Consumption and Infant Mortality (NSS 1987/88 and 2004/05)

Figure 1 illustrates two central points of our article. First, households that live in places with a greater burden of disease eat more calories, on average, despite their overall greater disadvantage. Second, changes in the disease environment can statistically account for much of the changes in calorie consumption over time. The graph plots the within-year associations between district IMR and household calorie consumption from cereals for each of the two time periods as non-parametric local regressions. Plotted over these lines are the annual averages of calorie consumption and IMR. The larger vertical distance between the dots is the full 200 cereal calorie decline; the smaller vertical distance between the lines indicates that at the same level of infant mortality the difference between the two time periods in cereal calorie consumption is small. Therefore, the within-year gradient between disease and consumption can statistically explain a visible fraction of the calorie decline.^13^

Table 2 reports estimates of (1), verifying the statistical robustness and significance of the associations documented in the Figure. Panels (*a*) and (*b*) are both included, for each regression specification, to verify that differences in demographic structure of households are not responsible for our results. For the main result in columns (1)-(7), the outcome variable is *per capita* calorie consumption of cereals; although we would expect a smaller effect on consumption further from energy needs requirements, column 8 substitutes total calorie consumption as a robustness check. Across columns, we add controls for heterogeneity across households in wealth or occupation. Overall, as shown by the consistently positive estimate in the first row, districts with the largest declines in IMR also observed the biggest drops in consumption of calories from cereals or from all food groups. The results suggest that improvements in the disease environment led to lower energy consumption.

**Table 2:** Larger Declines in Calorie Consumption in Districts that Experienced Bigger Reductions in Infant Mortality (National Sample Survey District Panel, 1987/88 to 2004/05)

Dependent variable:	D(1) Cereals	(2) Cereals	(3) Cereals	(4) Cereals	(5) Cereals	(6) Cereals	(7) Cereals	(8) Cereals	(9) All
Panel *(a):* **without detailed household demography controls IMR, district-level		4.340***	1.313***	0.975**	0.849*	1.203**	0.870*	0.811*	1.741**
	(0.397)	(0.496)	(0.472)	(0.478)	(0.569)	(0.476)	(0.479)	(0.724)
Round 61	-213.197***	-79.586***	-174.209***	-214.514***	-242.329***	1.192	-193.798***	-195.347***	-75.605***
	(10.236)	(15.548)	(18.544)	(17.885)	(22.271)	(23.223)	(17.895)	(17.994)	(26.243)
Panel (*b*): with detailed household demography controls IMR, district-level		4.661***	*\* 959***	0.974**	0.823*	1.151**	0.865*	0.798*	1.504**
		(0.401)	(0.482)	(0.463)	(0.467)	(0.559)	(0.466)	(0.468)	(0.686)
Round 61	-225.040***	-85.491***	-197.387***	-224.589***	-258.015***	15.865	-198.868***	-201.442***	-128.781***
	(10.585)	(15.903)	(18.385)	(17.806)	(22.035)	(22.863)	(17.684)	(17.817)	(25.600)
District fixed effects			X	X	X	X	X	X	X
In (real MPCE) (1987/88 Rs)				X	X	X	X	X	
Urban residence				X	X	X	X	X	
District average MPCE					X				
State-specific time trends						X			
Household controls							X	X	
Occupational controls								X	
Observations	228,234	228,234	228,234	228,234	228,234	228,234	228,234	228,234	228,234

*Notes.* Standard errors in parentheses are clustered at the district level (408 clusters). Infant mortality rate is number of children who die before reaching 12 months per 1,000 live births. ‘Cereals’ refers to rice, wheat, coarse grains, cereal substitutes, and the products of these items. The dependent variable is *per capita* calories consumption of either cereals or all food groups and is adjusted for meals eaten away from home. ‘Round 61’ is an indicator that equals 1 if households surveyed in 2004/05. MPCE stands for monthly *per capita* expenditures and was deflated by the CPIAL (rural households) or CPIIW (urban households) to 1987/88 Rupees. Household controls include indicators for: scheduled caste, scheduled tribe, Muslim, other religion (non-Hindu and non-Muslim), no literate female adult in household, and no literate male adult in household. Occupational controls are composed of 34 categories based on NCO-1968 codes. Households in the top and bottom 1% of [staple or all] calories distribution for each sector-round are dropped from the estimating sample. ***p < 0.01, **p < 0.05, *p < 0.10.

In column (1), we replicate the basic puzzle: daily *per capita* calories consumption of cereals fell by 213 kcal over 17 years. With the inclusion of IMR in the regression in column (2), we see that the unexplained gap is reduced by 63% (62% in panel (b) with demographic controls), although it remains statistically significant. Households living in districts with higher IMR consumed more calories from cereals, and more calories overall. The positive sign on IMR is important: if high infant mortality is a marker for a poor disease environment and if wealthier areas generally have greater access to food, health care, and public services like sanitation, then the estimate would seem to go in the ‘wrong’ direction - in the absence of the effect that this article documents - because it implies that households in poorer disease environments eat more calories.

With fixed effects, we identify the coefficient on IMR (β_1_) from district trends rather than levels; without these fixed effects, the model may be mis-specified to estimate a causal effect. Indeed, the magnitude of the estimate decreases, suggesting that crosssectional differences partially account for variation in calorie consumption. Once district fixed effects are added in column (3), including further controls - such as household consumption, urban residence, average district consumption and 34 occupation groups - changes the coefficient very little. In other words, all specifications based on within-district changes indicate that an additional infant death per 1,000 live births is associated with each person eating about one more calorie per day, on average.^14^ Notably for our empirical strategy, adding state-specific linear time trends in column (6) does not reduce the coefficient estimate, suggesting that our result is not due to heterogeneity in spurious secular trends. Similarly, column (5) verifies that the result is unchanged when district-level average MPCE is added as a control, which would account for any equilibrium effects due to differences in district-level prices, which is important to verify because our independent variable varies at the district-year level.^15^ These results suggest that improvements in the disease environment can account for some of the Indian calorie decline puzzle; in Section 5, we will combine these estimates from others in the article to assess the fraction of the decline that disease can explain.

## 3 Evidence from Local Disease Environments

We have seen that the districts where we observe greater improvements in the disease environment also experienced larger declines in average calorie consumption. This Section exploits comparative advantages of the 2005 India Human Development Survey (IHDS) (Desai *et al*., 2009).

Although the IHDS is a cross-section - therefore, we cannot directly study changes over time - this disadvantage is balanced by three advantages that complement the panel analysis of NSS data in the previous Section. First, the IHDS includes anthropometric measures of nutritional status for ever-married women 15-49 years old, children less than 5 years old and children 8-11 years old. Second, the IHDS permits a wider range of controls for economic, social, demographic, and occupational characteristics of households. Third - and perhaps most importantly - we can use the IHDS to compute local measures of the disease environment by matching households to survey primary sampling unit (PSU) level estimates of infant mortality and sanitation coverage. Districts in India, studied in the previous Section, are very large and contain much heterogeneity; PSU-level explanatory variables will more accurately capture the local disease environment to which households are frequently exposed.

This Section proceeds in four parts. First, subsection 3.1 documents that households consume more calories if exposed to a larger fraction of local neighbours who defecate in the open, even controlling for overall consumption and a range of social and demographic factors. Next, subsection 3.2 replicates this result and that of the previous Section by showing that households living in PSUs with more local infant mortality consume more calories. Then, subsection 3.3, presents a falsification test of the association between local morbidity and calorie consumption, showing that people who live near children with diarrhoea eat more but that other types of disease predictably have no effect. Finally, in a check of the plausibility of a nutritional effect of the disease environment, subsection 3.4 demonstrates that adult women exposed to more open defecation have lower body mass index (BMI), on average, even after accounting for household expenditure and calorie intake.

### 3.1 Effect of Sanitation on Calorie Expenditure

Do households exposed to more open defecation at the village or city sub-block level consume more calories on average? Exposure to germs in faeces may cause diarrhoea and other intestinal disease that diverts food from nutritional uses.

#### 3.1.1 Empirical strategy

The empirical strategy of this Section compares households exposed to different levels of local area open defecation at a fixed point in time. In particular, as an explanatory variable, we compute the fraction of households that defecate in the open instead of using a toilet or latrine for each PSU.^16^ We estimate the association of this variable with calorie consumption as follows:

(2) $$\begin{array}{rl} calories_{ip}= & \beta_{0}+\beta_{1}local\;open{\mathrm{defecation}}_{p}+\beta_{2}household\;open\;defecation_{ip} \end{array}+\beta_{3}\ln(MPCE{)}_{ip}+\beta_{4}urban_{p}+D_{ip}\theta_{1}+Y_{ip}\theta_{2}+S_{ip}\theta_{3}+E_{ip}\theta_{4}+\varepsilon_{ip},$$

where *i* indexes individual household and *p* indexes survey PSUs. As in subsection 2.2, we show that the results are robust to using either total calorie consumption or cereal calorie consumption as the outcome variable. The explanatory variable of interest is local area open defecation, a percentage from 0 to 100. An indicator for a household’s own open defecation is further included; this focuses the analysis on sanitation externalities while controlling for any average wealth difference between households who do and do not safely dispose of faeces.

Controls are added in stages to demonstrate robustness. In addition to household monthly consumption *per capita* and an indicator for urban residence, four vectors of controls are added. Demographic variables ***D*** are household size and the number of children in the household. Income sources, ***Y*** are as assigned by the IHDS into eleven categories: cultivation, allied agriculture, agricultural labour, non-agricultural labour, artisan, petty trade, business, salaried, professional, pension/rent, and others. Social groups S classifies households into one of eight groups: Brahmin, other higher castes, other ‘backwards’ castes, Dalit, Adivasi (or ‘tribal’), Muslim, Sikh or Jain and Christian; such social groups have been shown to be correlated with sanitation behaviour (Lamba and Spears, 2013). Finally, education E is a set of indicators for the highest education level of an adult in the household and an indicator for having at least one literate household member.

As a robustness check, we also include a specification where we control for local variation in the prices of rice and wheat, as reported to surveyors by interviewed households. These controls may not belong in a well-specified model: if a worse disease environment indeed increases demand for food at all prices, then disease will endogenously cause an increase in price. (Of course, we do not claim that any of our regression controls are randomly assigned, only that disease could endogenously influence food prices.) That said, we include a specification with this control to verify that the result does not change. Similarly, we control for PSU average monthly *per capita* expenditure and find similar results.

#### Results

**Fig. 2:**
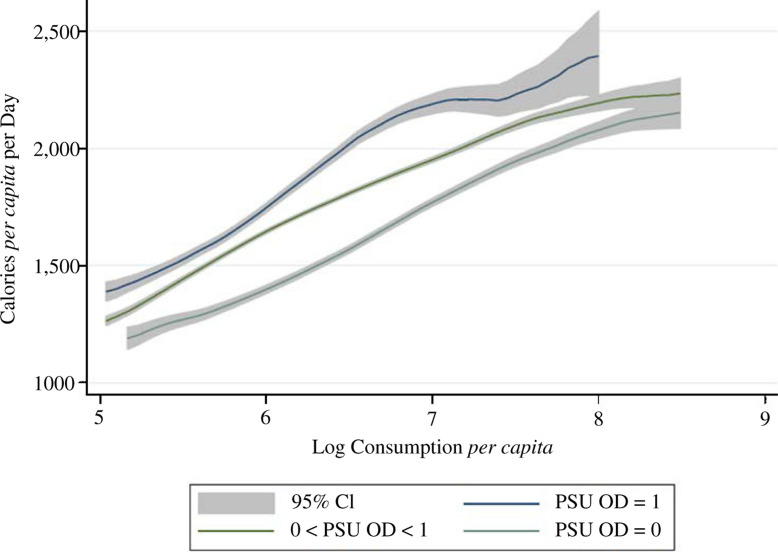
Calorie Consumption and Monthly Per Capita Expenditures by Local Sanitation Coverage (IHDS 2005)

**Fig. 3:**
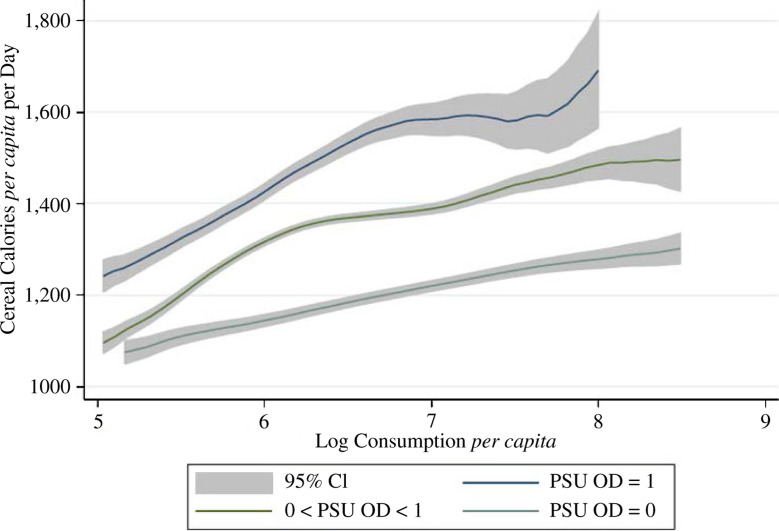
Cereal Calorie Consumption and Monthly Per Capita Expenditures by Local Sanitation Coverage (IHDS 2005)

Figures 2 and 3 provide initial evidence from the IHDS of a gradient between sanitation and total or cereal calorie consumption, respectively. Both graphs plot non- parametric regressions of average daily calories against overall household monthly consumption *per capita*. Not surprisingly, the graphs slope upwards, as richer households eat more. In both graphs, households are split into three categories according to the local disease externalities to which they are exposed: households in which no household surveyed in their PSU defecates in the open, households living in PSUs where all households defecate in the open and household living in PSUs at an intermediate level of open defecation. The graphs show that - at all levels of household consumption *per capita* - households exposed to more open defecation consume more calories, on average. The space between confidence intervals confirms that these differences are statistically significant and the gap at all levels of economic status suggests that the association between sanitation and calorie consumption does not merely reflect omitted wealth.

Are these differences robust to controls for demographic, income, social and educational characteristics of the household? Table 3 indicates that they are. As we add covariates to the regression model, the sanitation-calories gradient remains stable at about 1 calorie per percentage point of local open defecation exposure.^17^ To put this number in perspective, this would imply about a difference of 10 cereal calories per day associated with the 10 percentage point decline in open defecation in India between the 2001 and 2011 census rounds.

**Table 3:** Higher Average Calorie Consumption in Villages/Urban Sub-blocks with Lower Sanitation Coverage (India Human Development Survey, 2005)

	(1)	(2)	(3)	(4)	(5)	(6)	(7)
Panel *(a):* total calories							
Percentage of households without toilet in PSU	2.050***	1.663***	1 919***	1.649***	1.486***	0.580**	1.310***
	(0.256)	(0.276)	(0.283)	(0.283)	(0.272)	(0.259)	(0.309)
Household open defecation	-76.380***	84.173***	49.178***	45.216***	24.665**	39.60***	67.43***
	(13.778)	(12.286)	(12.335)	(12.006)	(11.221)	(11.97)	(12.10)
Log of MPCE		480.811***	409.943***	414.592***	440.704***	448.5***	460.4***
		(9.595)	(10.025)	(10.650)	(11.172)	(9.947)	(9.364)
Urban residence		-289.764***	-264.927***	-226.863***	-220.256***	-234.6***	-240.8***
		(18.786)	(19.045)	(19.459)	(18.673)	(16.50)	(19.40)
*N* (households)	39,673	39,673	39,673	39,673	39,673	39,570	39,673
Panel (*b*): cereal calories							
Percentage of households without toilet in PSU	2.304***	1.103***	1.332***	1 197***	1.060***	0.565**	0.438*
	(0.206)	(0.236)	(0.241)	(0.240)	(0.231)	(0.227)	(0.260)
Household opendefecation	36.811***	96.057***	68.131***	58.710***	31.183***	58.41***	95.22***
	(10.389)	(10.437)	(10.727)	(10.393)	(9.858)	(10.19)	(10.52)
Log of MPCE		178.018***	116.779***	129.576***	166.333***	179 3***	191 7***
		(7.691)	(8.332)	(8.873)	(9.280)	(8.433)	(7.813)
Urban residence		-266.414***	-246.726***	-217.579***	-205.830***	-204.5***	—211.0***
		(15.467)	(15.500)	(15.640)	(15.072)	(14.43)	(15.96)
*N* (households)	39,673	39,673	39,673	39,673	39,673	39,570	39,673
Demographics				X	X	X	X
Income sources				X	X	X	X
Social groups					X		
Education					X		
Cereal prices						X	
Average PSU (local area) MPCE							X

*Notes.* Standard errors in parentheses are clustered by PSU (2,473 clusters). The dependent variable is *per capita* calories consumption of either staple (cereals) or all foods. ‘Household open defecation’ means that the household has ‘no toilet/open fields’. MPCE stands for monthly *per capita* consumer expenditure and is measured in 2005 Rupees. ‘Demographics’ includes household size and number of children in households. For ‘Income sources’, the IHDS assigns households to one of 11 categories: cultivation, allied agriculture, agricultural labour, non-agricultural labour, artisan, petty trade, business, salaried, profession, pension/rent, and others. ‘Social groups’ classifies households into one of 8 groups: Brahmin, High Caste, OBC, Dalit, Adivasi, Muslim, Sikh/Jain, and Christian. ‘Education’ consists of highest educational attainment of adult 21+ years and of at least one literate adult in household. Households in top and bottom 1% of *per capita* calories distribution are dropped from the estimating sample. Please see Appendix Table 2 for further results with extended demographic controls. ***p < 0.01, **p < 0.05, *p < 0.10.

As before, this result may appear surprising because poorer people are more likely to defecate in the open and richer people eat more calories on average. This can be seen in the flip of the sign of household open defecation from statistically significantly negative in column (1) of panel (*b*) to statistically significantly positive in column (2), once the more precise control for household economic status is added. Additionally, the quantitative robustness of the main result to the sets of controls suggests that our finding is not a spurious reflection of heterogeneity in socio-economic status or work requirements.

#### 3.2 Effect of IMR on Calorie Expenditure

Can the results of Section 2 be replicated using local, i.e. village or urban sub-block level, infant mortality rates computed using IHDS in place of district IMR in the Indian census? In this subsection, we use the IHDS to estimate (2) from subsection 3.1 with infant mortality substituted for sanitation as the key explanatory variable. In particular, we compute the fraction of live births who reportedly died before their first birthday, linearly scaled as a count of deaths per 1,000 live births, for each survey PSU.^18^

Table 4 presents the results. The estimates are generally quantitatively consistent, albeit smaller in magnitude, with the district-level fixed effects results in Table 2; an extra infant death per 1,000 births is associated with 0.3 to 1.0 more calories consumed by each person each day. The smaller coefficient may reflect attenuation, since local IMR is computed from a small intra-PSU sample. Our preferred specification, column 3, includes all of the controls from Table 3. As a step towards replicating the district- level results, column (4) adds state fixed effects. Although we believe this is likely overcontrolling because the disease environment importantly varies at the state level, we include it for robustness and note that a statistically significant gradient remains. Column (5) shows that IMR and local sanitation are both predictors of calorie consumption when included together, which is consistent with a multidimensional disease environment (Coffey *et al*., 2013*b*). Finally, reiterating our concerns about the endogenous determination of prices, column (6) includes controls for cereal prices and finds coefficients qualitatively consistent with the other results.

**Table 4:** Higher Average Calorie Consumption in Villages/Urban Sub-blocks with Higher Infant Mortality (India Human Development Survey, 2005)

	(1)	(2)	(3)	(4)	(5)	(6)
Panel (*a*). cereal calories						
Infant mortality rate,	0.924***	0.799***	0.648***	0.278**	0.554***	0.394**
PSU-level	(0.143)	(0.141)	(0.142)	(0.124)	(0.147)	(0.131)
Percentage of					0.878***	
households without					(0.239)	
toilet in PSU						
Household open					32.308***	
defecation					(9.821)	
Log of MPCE		148.491***	156.392***	194.493***	166.729***	205.7***
		(7.982)	(9.066)	(8.116)	(9.205)	(8.759)
Urban residence		-331.257***	-240.138***	-178.206***	-207.715***	-179.5***
		(13.077)	(13.643)	(11.055)	(15.054)	(12.45)
Price of wheat (Rs/kg)						-26.56***
						(2.552)
Price of rice (Rs/kg)						-28.30***
						(1.574)
*N* (households)	39,652	39,652	39,652	39,652	39,652	39,652
*Panel (b): Total calories*						
Infant mortality	0.768***	1.017***	0.832***	0.349***	0.711***	0.426**
rate, PSU-level	(0.141)	(0.152)	(0.152)	(0.130)	(0.155)	(0.134)
Percentage of					1.249***	
households without					(0.280)	
toilet in PSU						
Household open					26.357**	
defecation					(11.217)	
Log of MPCE		448.985***	429.049***	443.311***	441.223***	468.9***
		(9.659)	(10.955)	(9.741)	(11.104)	(10.66)
Urban residence		-369.829***	-265.138***	-201.027***	-223.038***	-201.5***
		(15.828)	(17.052)	(13.252)	(18.525)	(14.69)
Price of wheat (Rs/kg)						-61.20***
						(2.971)
Price of rice (Rs/kg)						-18.90***
						(1.957)
*N* (households)	39,652	39,652	39,652	39,652	39,652	39,652
Household controls			X	X	X	X
Extended household						X
demography						
State fixed effects				X		

*Notes.* Standard errors in parentheses are clustered by PSU (2,470 clusters). The dependent variable is *per capita* calories consumption of either staple (cereals) or all foods. Infant mortality rates are computed from IHDS. ‘Household open defecation’ means that the household has ‘no toilet/open fields’. MPCE stands for monthly *per capita* consumer expenditure and is measured in 2005 Rupees. Household controls are the exact same set of comeple household, educational, and social controls as used in Table 3. Extended household demography includes indicator controls for each number of persons, children, teens, married men and married women. Households in top and bottom 1% of *per capita* calories distribution are dropped from the estimating sample. ***p < 0.01, **p < 0.05, *p < 0.10.

Column (6) of Table 4 also adds controls for extended dimensions of household demography: indicators for counts of the numbers of married adult males and females, and for the count of teenagers. In the rural Indian context, it is in principle possible that married daughters-in-law might eat less than daughters of the village, for example (Jeffery *et al*., 1989). For complete consistency across Tables, Appendix Table A2 replicates the last three columns of Table 3 with these extended demographic controls added, and the results are unchanged.

#### 3.3 Falsification Test. Type of Disease and Calorie Consumption

Subsection 3.2 showed that households living in local areas with higher infant mortality rates consume more calories, on average; this result is informative because IMR is an important and widely used measure of disease environments but it is not specifically a measure of the type of disease most related to nutritional outcomes and needs. In this Section, we report a similar analysis using different explanatory variables: the fraction of children under 5 in a local area who have suffered from diarrhoea, fever, or cough in the last month, as reported by their mothers. If our results are indeed driven by enteric morbidity reducing the absorption and use of nutrients in food, then we would expect to see an association between calorie consumption and diarrhoea, but not for other causes of disease.

Table 5 presents the results. There is an economically large and statistically significant association between local diarrhoea and calories consumption but there is no association for fever or cough. A 10 percentage point increase in the fraction of a household’s neighbouring children suffering from diarrhoea is associated with an approximately 35 calories per person increase in daily consumption; estimates for fever and cough are of much smaller magnitude and are not statistically distinguishable from zero. These results are unchanged by controlling for household consumption, local area average consumption and the demographic structure of the household (indicators for each count of number of persons, children, teens, married males and married females). The specificity of this result is consistent with a causal effect of the enteric disease environment on calorie needs, which is precisely what would be most influenced by improvements in sanitation.

**Table 5:** Households Who Live Near Children with Diarrhea Eat more Calories, IHDS

	(1)	(2)	(3)	(4)
	*Per capita* daily calorie consumption			
Diarrhoea local fraction	348.2***	366.8***	346.0***	243.4***
	(68.29)	(71.62)	(70.83)	(59.37)
Fever local fraction	56.99	80.91	69.36	33.94
	(60.48)	(60.89)	(58.72)	(50.39)
Cough local fraction	8.104	-18.30	10.33	2.716
	(62.50)	(63.31)	(60.98)	(52.98)
Urban	-378.8***	-343.0***	292.4***	-192.0***
	(15.54)	(15.70)	(17.13)	(15.34)
Log of *per capita* household consumption	435.5***	366.8***	432.7***	471.5***
	(8.998)	(9.222)	(8.618)	(9.616)
Log of PSU average *per capita*			-165.2***	-66.29***
household consumption			(20.13)	(18.26)
Household size and composition controls		X	X	X
Complete controls from Table 4				X
*N* (households)	39,654	39,654	39,654	39,459

*Notes.* Standard errors clustered by survey PSU. Disease ‘local fractions’ are the fraction of children under 5 in the survey PSU who were reported by their mother to have had that symptom within the last month. Column (4) incudes every control (demographic, extended demographic, educational, social, and local rice and wheat prices) from Table 4, which uses the same data source. ***p < 0.01, **p < 0.05, *p < 0.10.

##### 3.3.1 Household-level disease

As throughout this article, this is an analysis of the local disease environment, including externalities. One advantage of this approach is that confounding omitted variables may be less relevant when considering the open defecation of a household’s neighbours. Additionally, externalities of the disease environment highlight the importance of sources of disease such as sanitation to public economics, as a potential policy issue (Geruso and Spears, 2015). This is consistent with a literature on disease in developing countries that has emphasised the role of village-level average open defecation or sanitation (such as in, for example, cluster randomised trials of latrine provision (Gertler *et al*., 2015) or deworming (Miguel and Kremer, 2004), rather than randomisation at the household level).

That said, we can also compare calorie consumption in households whose own members have more or less diarrhoea. A one standard deviation (9 percentage point) increase in the fraction of household members having diarrhoea in the last month (where diarrhoea is measured as residuals after detailed demographic controls for age structure and sex) is associated with the households consuming 43 more calories *per capita* per day, on average, controlling for log MPCE, urban residence and non- parametric sets of indicators for household size and for the count of children. This result may be in contrast with some evidence in the nutritional literature that children suffering from acute infections may consume less during the course of their illness (Stephensen, 1999). However, it is not our claim that acute diarrhoea in particular is the only or most important mechanism that links the disease environment to increased caloric intake needs; rather, one very important mechanism for which evidence is accumulating in the epidemiological and medical literature is chronic enteric disease due to repeated environmental exposure which would reduce the body’s ability to absorb and use nutrients.

### Effect of the Disease Environment on BMI

If the local disease environment increases calorie needs, then we might expect to see it reflected in measured nutritional status. Although height reflects early-life health and net nutrition and would not be expected to respond to the current disease environment, weight-for-height in contrast, reflects more recent net nutrition. Indeed, given the difficulty of meaningfully measuring diarrhoea morbidity with surveys, some researchers advocate using child weight as a proxy indicator of recent disease (Schmidt *et al*., 2011). Therefore, as a verification of the nutritional mechanism of our main results, we study the association between the local disease environment and the BMI of ever-married adult women, aged 20-45.

**Fig. 4:**
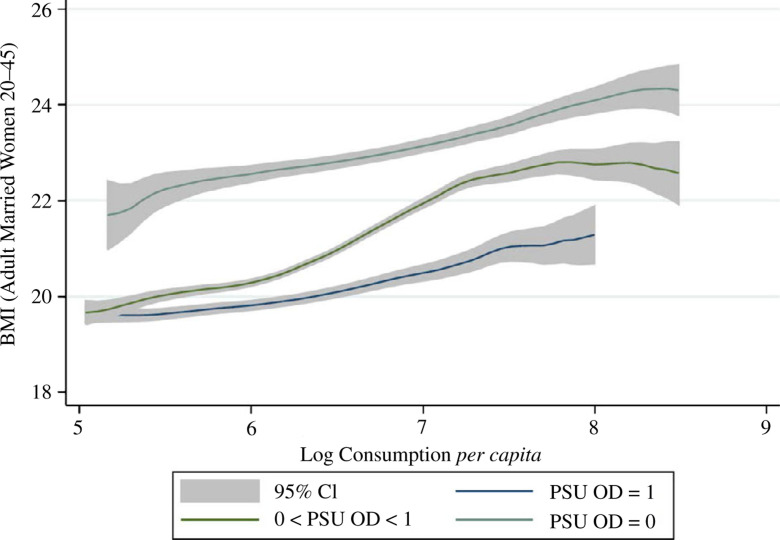
Adult Women’s BMI and Monthly Per Capita Expenditures by Local Sanitation Coverage (IHDS 2005)

**Fig. 5:**
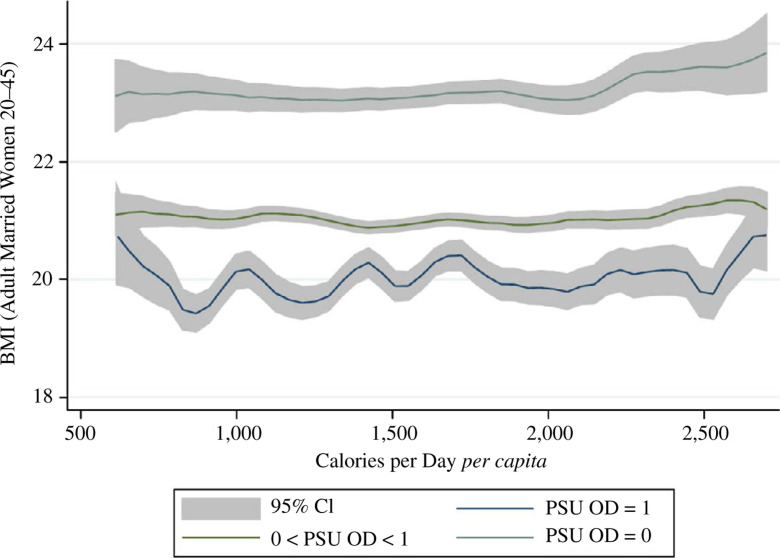
Adult Women’s BMI and Calorie Consumption by Local Sanitation Coverage (IHDS 2005)

Do women exposed to more disease externalities weigh less and, if so, do the differences in body size merely reflect economic status or consumption? Figures 4 and show an association between sanitation and average body mass across levels of household expenditure using the same sample-splitting strategy as in Figures 2 and 3. As Figure 4 unsurprisingly depicts, richer women in India weigh more, on average. However, at all levels of household consumption, women exposed to more local area open defecation weigh statistically significantly less. Figure 5 repeats this analysis, using household daily calorie consumption *per capita* in place of overall consumption. Strikingly, there is little apparent relationship between calorie consumption and women’s weight.^19^ As before, at all levels of household calorie consumption, women exposed to less open defecation weigh statistically significantly more.

Table 6 verifies the statistical significance and robustness of this result. Living in a PSU where nobody defecates in the open is linearly associated with being about one BMI point heavier, on average, than living in a PSU where everybody defecates in the open. This result is stable controlling for household overall and calorie consumption and for several vectors of controls. Column (7) demonstrates that the result is unchanged when the woman’s height is added as a further control; height is a marker of early-life well-being and is in the denominator of BMI, so this control verifies that a mechanical correlation with height is unlikely.^20^ These results are consistent with our overall interpretation of our findings: exposure to a more threatening disease environment increases calorie needs.

**Table 6:** Lower Average BMIAmongWomen Who Live in Villages/Urban Sub-blocks with Lower Sanitation Coverage (India Human Development Survey, 2005)

	(1)	(2)	(3)	(4)	(5)	(6)	(7)
Panel *(a):* BMI regressed on sanitation and cereal calories							
Percentage of households without toilet in PSU	-0.02993***	-0.02954***	-0.01903***	-0.01086***	-0.01118***	-0.00996***	-0.00968***
	(0.00096)	(0.00097)	(0.00124)	(0.00179)	(0.00170)	(0.00162)	(0.00160)
*Per capita* calories consumption: cereals		-0.00015**	-0.00032***	-0.00029***	-0.00024***	-0.00002	-0.00005
		(0.00007)	(0.00007)	(0.00007)	(0.00006)	(0.00007)	(0.00006)
Log of MPCE			1.01412***	0.91590***	0.79735***	0.28203***	0.35188***
			(0.05844)	(0.05645)	(0.05613)	(0.06330)	(0.06407)
Urban residence			0.44273***	0.47965***	0.39016	0.07605	0.09114
			(0.08661)	(0.08662)	(0.31245)	(0.30412)	(0.30087)
Household open defecation				-0.86832***	-0.78528***	-0.34951***	-0.35241***
				(0.12580)	(0.11550)	(0.10703)	(0.10778)
Height, in centimeters							-0.07480***
							(0.00593)
*N* (PSU)	2,452	2,452	2,452	2,452	2,452	2,452	2,452
*N* (adult women)	27,135	27,135	27,135	27,135	27,135	27,135	27,123
Panel (*b*): BMI regressed on sanitation and total calories							
Percentage of households without toilet in PSU	-0.02993***	-0.03017***	-0.01929***	-0.01095***	-0.01128***	-0.00998***	-0.00971***
	(0.00096)	(0.00096)	(0.00124)	(0.00180)	(0.00170)	(0.00162)	(0.00160)
*Per capita* calories consumption: Total		0.00020***	-0.00016***	-0.00014**	-0.00011**	0.00000	-0.00001
		(0.00005)	(0.00006)	(0.00006)	(0.00005)	(0.00005)	(0.00005)
Log of MPCE			1.03137***	0.92900***	0.80109***	0.27624***	0.34249***
			(0.06338)	(0.05979)	(0.05835)	(0.06461)	(0.06520)
Urban residence			0.48228***	0.51623***	0.42054	0.08030	0.09875
			(0.08606)	(0.08622)	(0.31285)	(0.30401)	(0.30089)
Household open defecation				-0.88278***	-0.79561***	-0.34843***	-0.35013***
				(0.12740)	(0.11659)	(0.10652)	(0.10730)
Height, in centimetres							-0.07470***
							(0.00592)
*N* (PSU)	2,452	2,452	2,452	2,452	2,452	2,452	2,452
*N* (adult women)	27,135	27,135	27,135	27,135	27,135	27,135	27,123
Income source					X	X	X
Household controls						X	X

*Notes.* Standard errors in parentheses re clustered at the PSU level (2,452 clusters). PSU stands for ‘primary sampling unit’, which is typically a village or city sub-block. The dependent variable is BMI or body mass index. Sample primarily consists of married women between 18 and 49 years old. Household controls include main income source, household assets index, social group, household size, number of children in household, and any literate adult in household. ***p < 0.01, **p < 0.05, *p < 0.10.

## 4 Evidence from Detailed Occupational Data

We have shown an effect of the disease environment on calorie consumption using variation in the disease environment within district over time and variation across villages or city sub-blocks in the cross-section. On average, households in worse disease environments consume more calories from cereals and all food groups. As noted by Deaton and Drèze (2009) and explored in detail by Eli and Li (2013), one candidate explanation for India’s calorie decline is the reduction in energy requirements for work. Both lower work requirements and an improving disease environment are likely to have contributed to India’s calorie decline and this Section asks whether changing patterns of work are an omitted variable in our estimates that drives the effect of the disease environment.

The analyses in Sections 2 and 3 both included controls for urban or rural residence and for the primary occupational category of the household. We now turn to the NSS 1983 cross-section because the survey collected information on calorie consumption, local sanitation coverage and highly detailed occupational categories for each household. Our main finding is that, even conditional on detailed controls for work, along with MPCE and household characteristics, households from villages or urban sub-blocks with worse sanitation consumed more calories from cereals and from all food groups. This suggests that, although differences in energy needs for income- generating activities may be independently important, they are not responsible for our results.

### 4.1 Empirical Strategy

In NSS Round 38 (1983), the same households were interviewed for the CES and EUS. In addition, households reported whether they use a toilet or latrine.^21^ The dataset does not have district identifiers and, therefore, we cannot link it to our district panel nor can we match district-level IMR from the Census. Instead, as in Section 3, we compute PSU-level^22^ sanitation coverage as a measure of local sanitation conditions to proxy for the disease environment.

Sample statistics are reported in column (4) of Table 1. Note that NSS Rounds 38 (1983) and 43 (1987/88) are similar according to the measures listed in the Table. *Per capita* calories consumption of cereals and all food groups are 1,564 and 2,140 calories per person per day, which are approximately 14 calories higher and 32 calories lower than the averages in Round 43 respectively. Poverty is clear in the data: nearly 80% of households do not have a toilet, and food expenditures are almost 70% of the household budget.

We estimate the regression equation:

(3) $$\begin{array}{rl} ca\left|\right|lories_{ips}= & \alpha_{0}+\alpha_{1}local\;open{\mathrm{defecation}}_{ps}+\alpha_{2}household\;open\;defecation_{ips} \end{array}+\alpha_{3}ln(MPCE{)}_{ips}+\sum_{j=1}^{J}\omega_{j}\;employment_{ips}^{j}+X_{ips}\vartheta +\zeta_{s}+\varepsilon_{ips},$$

where *i* is an index for household, *p* for PSU (i.e. village or urban sub-block), and s forstate- region. There are 77 state-regions in NSS Round 38, and state-regions are contiguous districts grouped by geographic features, population densities, and cropping patterns. The key explanatory variable is the percentage of households in the PSU without a latrine (local open defecation). We also control for whether the individual household has its own (or shares) a latrine and for the household’s monthly *per capita* expenditures in logarithms. Additional covariates (X*ips*) include household’s caste, religion, type of flooring, source of drinking water, and type of cooking fuel. Flooring, water source, and cooking fuel proxy for household socio-economic status and access to public goods.

Importantly, we control for work requirements in two ways. First, we control for demographic-specific work variables. We calculate the share of household members who fall into one of 38 age-sex-industry categories. There are seven age categories and five industry categories.^23^ Second, we control for the household’s primary occupation, which are using 3-digit NCO-1968 codes. In NSS 1983, there are 665 groups. The first method accounts for differences across households in demographic composition as well as the type of work across members within the household. The second method models the household as a single unit. To the extent that member-specific employment information might be noisy, the primary household-level occupation may be a preferred indicator. The results of both methods are shown in Table 7. With the inclusion of one or both of these work controls, identification of the effect of local sanitary conditions (captured by α_1_) comes from variation within state-region, across PSUs, holding constant household wealth and differences in occupation types, industries, or demographic composition.

**Table 7:** Higher Average Calorie Consumption in Villages/Urban Sub-blocks with Lower Sanitation CoverageConditional on Detailed Demography-specific Household Employment Information (National Sample Survey Round 38, 1983)

	(1)	(2)	(3)	(4)	(5)	(6)	(7)	(8)
Dependent variable	Cereals	Cereals	Cereals	Cereals	Cereals	Cereals	All	All
Percentage of households	5.431***	3 944***	2.405***	1.266***	1.380***	1.206***	1.639***	1.186***
without toilet in PSU	(0.128)	(0.271)	(0.258)	(0.240)	(0.242)	(0.235)	(0.275)	(0.256)
Log of MPCE		403.525***	475.928***	555.460***	509.428***	521.768***	1,046.001***	1,005.566***
		(8.083)	(8.107)	(7.193)	(7.682)	(7.493)	(7.531)	(8.007)
Urban residence		-341.163***	—271 747***	-167.560***	-175.736***	-149.592***	-296.680***	-165.460***
		(17.833)	(17.634)	(15.073)	(15.765)	(14.812)	(18.684)	(16.561)
Household controls			X	X	X	X	X	X
State-region fixed effects				X	X	X	X	X
Demography-specific				X		X		X
employment controls								
Household primary					X	X		X
occupation controls								
*N* (PSUs)	2,991	2,991	2,991	2,991	2,991	2,991	2,991	2,991
*N* (households)	108,330	108,330	108,330	108,330	108,330	108,330	108,098	108,098
R^2^	0.064	0.188	0.225	0.356	0.370	0.386	0.518	0.562

*Notes.* Standard errors in parentheses are clustered by PSU (2,991 clusters). PSU stands for ‘primary sampling unit’, which is typically a village or city sub-block. ‘Cereals’ includes rice, wheat, coarse grains, cereal substitutes and the products of these items. The dependent variable is *per capita* calories consumption of either cereals or all food groups and is adjusted for meals eaten away from home. Household controls include: scheduled tribe, scheduled caste, Muslim, other religion (non-Hindu and non-Muslim), mud flooring, open water source, and dirty cooking fuels. Demography-specific employment controls are the percentage of household members who fall into sex-age-industry (based on principal industry division for individuals 15+ years old) category. Household occupation controls include indicators for 3-digit NCO-1968 codes (665 groups), indicator for agricultural industry, and indicators for crops cultivated. MPCE stands for ‘monthly *per capita* expenditures’, which is measured in 1983 Rupees. There were 77 NSS state-regions at the time of survey; state-regions are continguous districts grouped by geographical features, population densities, and cropping patterns. Households in top and bottom 1 % of *per capita* calories distribution in the rural and urban sectors were trimmed before estimation. ***p < 0.01, **p < 0.05, *p < 0.10.

### 4.2 Effect of Sanitation on Calories Conditional on Work

If the association of sanitation on calorie consumption was largely driven by spuriously correlated differences in energy needs for work activity, then once we controlled for the household’s primary occupation, there would be no systematic relationship between latrine coverage and calories; in other words, α_1_ would fall to zero. The results in Table 7 reject this hypothesis and show that energy needs for work requirements do not explain why households in worse disease environments consume more calories per person.

Across the columns of Table 7, we include different combinations of control variables to predict *per capita* calories consumption of cereals (columns (1)-(6)) or all food groups (columns (7)-(8)). Without any control variables, we find that there is a strong positive relationship between local latrine coverage and calorie consumption; however, this coefficient is unlikely to represent a causal effect. Once we condition on MPCE, urban residence and state-region fixed effects, the estimate decreases in magnitude but remains strongly significant. Moreover, it is quantitatively comparable to our earlier estimates from different empirical strategies.

How much of the apparent effect of sanitation on calorie consumption might actually reflect differences related to work? Juxtaposing columns (4) and (5) or columns (4) and (6), we see that there is not a large change in the coefficient estimate whether we control for state-region fixed effects or work variables, as captured by 38 demography-specific industry categories or 665 primary occupational categories. Including all work-relevant characteristics as explanatory variables in the regression (column (6)), we still find that local sanitation is a strong predictor of higher calories consumption. Relative to a person from a village where no one defecates in the open, a person from a village where 80% ofhis or her neighbours defecate in the open consumed an additional 100 calories per day, on average. For calories from all food groups in columns (7)-(8), we confirm that our main result is robust to controls for detailed work controls along with MPCE and household characteristics. Because these detailed work controls do not importantly change our estimates - either within this data set, or in comparison with the estimates in Sections 2 and 3 - it is unlikely that changes in work requirements are responsible for our results.

## 5 How Much of the Calorie Decline Could the Disease Environment Explain?

Two empirical strategies using three separate datasets find quantitatively similar effects of the disease environment on calorie consumption in India. In light of these estimates, how much of the Indian calorie decline could be statistically accounted for by an improving disease environment? This Section uses two complementary methods to estimate the fraction of the gap that can be explained.

First, we apply the regression results of this article to predict linearly the change in calorie consumption associated with the observed change in the disease environment. Therefore, we calculate:

(4) $$percent\;explained=\hat{\beta }\frac{∆disease}{∆calories}.$$

The disease-calories gradient ß^ is taken from various estimates from the regression Tables in this article. For completeness, we separately use change in IMR and change in sanitation and change in cereal and total calorie consumption, all taken from Table 1 of summary statistics.

Results are reported in panels (*a*) and (*b*) of Table 8. Both the change in infant mortality and the change in sanitation linearly predict a decrease in calorie consumption that would account for about 20% of the gap or more. To the extent that the changes in and effects of sanitation and IMR are independent of one another, the true total percentage explained by improvements in the disease environment may be even greater.

**Table 8:** How Much of the Calorie Decline can an Improving Disease Environment Account for ?

Table	Column	Outcome	Slope	Percentage explained
Panel (a): IMR gradient				
2	9	All	1.741	43
2	9*	All	1.504	37
2	3	Cereal	1.313	32
2	3*	Cereal	1.252	31
2	6	Cereal	1.203	30
2	6*	Cereal	1.151	28
4	2	All	1.017	25
2	4*	Cereal	0.974	24
2	5*	Cereal	0.823	20
4	2	Cereal	0.799	20
4	6	All	0.426	10
4	6	Cereal	0.394	10
Median percentage				27
Panel (*b*): Sanitation gradient				
6	3	Cereal	1.694	27
6	7	All	1.639	26
3	5	All	1.486	23
6	6	Cereal	1.206	19
6	8	All	1.186	19
3	5	Cereal	1.060	17
Median percentage				21
Panel (c): Decomposition of calorie change due to IMR, NSS panel				
Oaxaca-Blinder, equal weight		Cereal		82
Oaxaca-Blinder, pooled		Cereal		87
Non-parametric reweighting		Cereal		77
Oaxaca-Blinder, equal weight		All		23
Oaxaca-Blinder, pooled		All		51
Non-parametric reweighting		All		44

*Notes.* As in Table 2, decomposition analysis in panel (c) omits the top and bottom 1% of households. *Extensive demography controls included.

Next, panel (*c*) presents results from econometric decomposition analyses. These estimate the fraction of an average difference in an outcome between two groups that can be accounted for by differences in observable characteristics (Spears, 2012a). Like any other analysis of observable data, a causal interpretation of a decomposition depends on the nature of the heterogeneity in the explanatory variables; decompositions such as these may overestimate the fraction causally explained if the associations that they use include omitted variable bias.

We use Oaxaca (1973)-Blinder (1973) decompositions that apply a similar linear method to that which is shown in (4). Different decomposition methods construct different estimates of the slope *β^*. For robustness, we use two different estimates of *β:* the simple regression slope from the pooled data, and an equally weighted average of the slopes estimated from within the two survey rounds, or points in time.

Additionally we use a non-linear decomposition that non-parametrically reweights the sample from the 1980s to match the distribution of exposure to disease in the sample from the 2000s sample (DiNardo *et al*., 1996) .^24^ In particular, we divide each sample into 16 infant mortality ‘bins’ *b* corresponding to intervals of 5 infant deaths per 1,000 live births. We then compute for each bin and each sample $w_{b}^{43}$ and $w_{b}^{61}$ the fraction of the sample in the 43rd and 61st survey rounds that are in bin *b*, using household survey weights. Each observation *i* in the 43rd round is given a new weight:

(5) $${\tilde{\pi }}_{i}=\pi_{i}\frac{\omega_{b\left(i\right)}^{61}}{\omega_{b\left(i\right)}^{43}},$$

where π_*i*_ is the survey weight of household *i* computed by the NSS and *b(i)* is the IMR bin to which household *i* belongs. Finally, we compute a counterfactual calorie consumption mean for the 43rd round, if it had had the same distribution of exposure to IMR as the 61st round, as:

(6) $$\;counterfactual\;calories^{43}=\frac{\sum_{i}{\tilde{\pi }}_{i}\;calories_{i}^{43}}{\sum_{i}{\tilde{\pi }}_{i}}.$$

Panel (*c*) of Table 8 presents the results of these three decomposition methods, applied to the change between the 43rd and 61st NSS rounds in calorie consumption and cereal calorie consumption. Unlike the decomposition approaches in panels (*a*) and (*b*), these results are not based on causally narrow effect estimates. Nevertheless, changes in the disease environment over the two decades studied can statistically account for a substantial fraction of the decline in calorie consumption over this period. These results are consistent with our earlier observation that - if IMR and sanitation represent at least partially non-overlapping dimensions of the disease environment - ‘over 20%’ may be only a lower bound on the part explained. However, no estimate here of the percentage explained suggests that the disease environment can account for the entire calorie decline.

## 6 Conclusion

Over the past several decades, average calorie consumption in India has declined substantially. In this article, we have presented and assessed the evidence for one candidate explanation: a gradually improving disease environment. Two complementary empirical strategies applied to different datasets estimate robust and quantitatively comparable effects of the disease environment on average calorie consumption. These estimates suggest that the disease environment could account for at least one-fifth of India’s recent calorie decline.

Because India still faces an important burden of preventable infectious disease, these estimates suggest that the Indian economy may suffer a large cost of wasted calorie consumption. Any computation of such a cost is highly approximate. With this strong caveat, taking our linear regression results literally suggests that reducing open defecation rates in India from over 50% to zero could reduce *per capita* calorie needs by about 50 calories per day. If a poor person’s calories have a marginal cost of about 0.02 cents apiece (1.25 international dollars of total consumption per day, with one third on food, and 2,000 calories), then eliminating open defecation would save about four dollars per person per year in food consumption, which is about one-tenth of one percentage point of GDP *per capita*. Again, there are many approximations in this figure: for example, some well-nourished people may feel no effect; different people have differently priced marginal calories; and this computation ignores potential improvements in other dimensions of the disease environment.^25^ We include it merely to suggest that effects on calorie needs of the disease environment could add up to an important economic cost.

## Figures and Tables

**Fig. A1:**
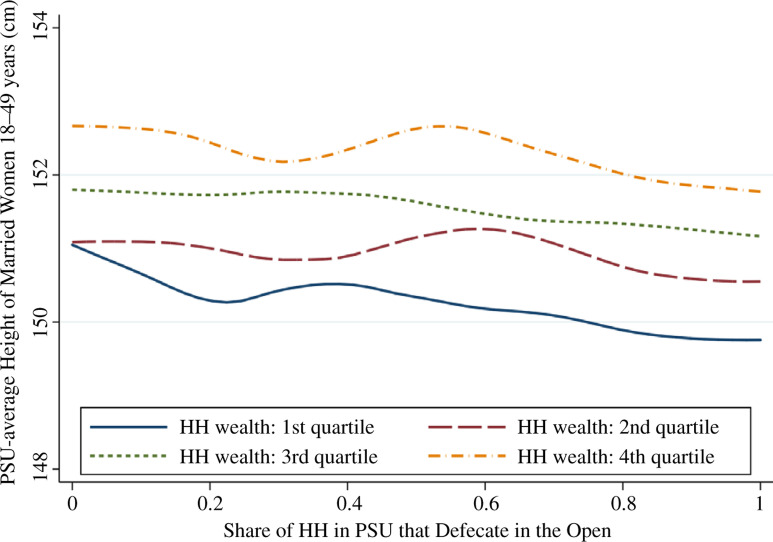
Adult Women’s Height and Local Sanitation Coverage, by Household Socioeconomic Status Note. HH wealth is measured in monthly consumption per capita.

## Footnotes

1 ‘Food consumption’ refers to caloric intake as opposed to food variety.

2 Cereals refer to: rice/rice products, wheat/wheat products, jowar/jowar products, bajra/bajra products, ragi/ragi products, maize/maize products, small mil lets/small millets products, and barley/barley products.

3 The 1981 Census of India did not measure rural sanitation because rural open defecation was almost universal.

4 GDP *per capita* (in constant 2,000 US dollars) was $320.92 in 1983 and $740.11 in 2005 (World Development Indicators, World Bank). The average annual compounded growth rate (*r*) is calculated as 740.11 = 320.92(1 + r)^(2005-1983)^).

5 Moreover, despite the correlation of height in India with human capital (Spears, 2012b), differences in economic growth across Indian states have not been associated with reductions in child malnutrition (Subramanyam et *al*., 2011; Coffey *et al*., 2013a).

6 Disease externalities could also contribute towards explaining Logan’s (2006) finding that the expenditure elasticity of calorie was greater for British than for American households in the late nineteenth century, if population density was greater in Great Britain.

7 Scrimshaw *et al.* (1968, p. 59) write: ‘Infections so consistently worsen nutritional status that they must be taken into account in all clinical problems and public health programmes that involve persons whose diet is inadequate or whose nutritional status is suboptimal.’

8 There are ‘thick’ and ‘thin’ rounds that correspond to the survey sample size: ‘thick’ rounds, which include approximately 120,000 households in each survey, serve as the basis of India’s consumption and poverty statistics, whereas ‘thin’ rounds, approximately 40,000-50,000 households, help to monitor trends in- between ‘thick’ rounds. Both Rounds 43 and 61 are ‘thick’ rounds and, in our study, we use the CES to look at household food and non-food expenditures.

10 We deflate nominal monthly *per capita* expenditures by the Consumer Price Index for Agricultural Labourers in rural areas (by month and year) and Consumer Price Index for Industrial Workers in urban areas (by month and year).

11 For robustness, we also conducted the analysis after aggregating to the district level, i.e. regress mean *per capita* calorie consumption on infant mortality rates and log of the district average monthly *per capita* expenditures with district-level observations. To ensure that our results are not driven by outliers, we trim the sample for districts with mean calorie consumption levels in the far upper and lower tails (top and bottom 1% by survey round). In Appendix Table A1, we show the results with a balanced panel, i.e., only districts with observations in both rounds; this amounts to trimming approximately 6% of districts with non-missing values for IMR. The coefficient estimates for IMR are consistent with Table 2, and F-tests show that we cannot reject that the estimates are equal to 1.

12 The set of age ranges are: 0-4 years old, 5-9 years old, 10-14 years old, 15-39 years old, 40-49 years old, 50-59 years old, and 60 or more years old.

13 We note that the level of cereal calorie consumption is steady around 1,400 calories per person per day for households living in districts with IMR exceeding 70 deaths per 1,000 live births in 2004/05. The cereal share of total calorie consumption is also fairly flat in the range of 70-5% in these districts in 1987/88 and in 2004/05.

14 We cannot reject that β_1_ = 1 in each specification represented in columns (3)-(8).

15 Our main results are the outcome with cereal calories; in this case, the coefficient on IMR is 0.849 as shown in column 5 of panel (*a*). Although not reported in the Table, if we compute the same regression, including the control for district average MPCE but with all calories as the dependent variable, the coefficient on IMR becomes 0.775; this result is not statistically significantly different from the cereal calories result, but with a large standard error of 0.61 its confidence interval includes zero.

16 Spears (2013) and Kov *et al.* (2013) have shown in various contexts that this variable is associated with child height-for-age, which is commonly cited as an indicator of nutritional status.

17 This point estimate is smallest in column (6), but a F-test does not reject a null hypothesis that the coefficient is 1 even with this smaller estimate.

18 On the one hand, this specification reduces measurement error in IMR exposure, relative to the district- level analysis, by matching the exposure more narrowly to a household’s local environment. On the other hand, this specification increases measurement error by estimating each IMR statistic from a smaller sample. If the increase in measurement error dominates, this would cause attenuation bias, which would bias our estimate of the effect of IMR on calorie consumption towards zero.

19 Deaton and Dreze (2009) note that declining calorie consumption in India coexists with small improvements in child nutritional status. Spears *et al.* (2013) show that although there is a relationship between district level open defecation and child stunting rates in India, stunting rates are uncorrelated with district calorie consumption averages. We also observe that *household* calorie consumption may be a poor measure of the calorie consumption of married women of childbearing age, who often suffer low intrahousehold status in India (Coffey *et al*., 2013c).

20 As a falsification test, we examine whether local sanitation coverage predicts women’s heights, conditional on household wealth. Because most women in India move to their husband’s family’s home upon marriage, many women may face a different disease environment as adults than they did as children. As shown in Appendix Figure 1, there is little correlation between height and contemporaneous rates of open defecation. This non-result is reassuring because adult height - as a marker of early-life health and cumulative health shocks (Case and Paxson, 2008) - should not systematically vary with current sanitation coverage unless healthier people are selected into better environments, even after accounting for differences in wealth.

21 The analysis linking sanitation, calories consumption and employment can only be done with this ‘thick’ survey round. The question about household toilet use was dropped from future questionnaires for these surveys. After Round 50 (1993/94), the CES and EUS were no longer administered on the same set of households because the surveys took too much time.

22 In the NSS, the Primary Sampling Unit (PSU) is a village for the rural sector and block for the urban sector. If the population per village/block exceeds one million inhabitants (i.e. 10 lakhs), then the unit is further divided into hamlet groups or sub-blocks.

23 The seven age categories are: (1) 0-4 years, (2) 5-9 years, (3) 10-14 years, (4) 15-39 years, (5) 40-59 years, (6) 60-99 years and (7) missing age. The five industry categories are: (1) principal industry in agriculture/mining/construction; (2) principal industry in manufacturing/electricity/trade/business; (3) principal industry in services; (4) principal working status as domestic duties/unemployed/other/in school/too young or too old and subsidiary industry in agriculture/mining/construction/manufacturing/electricity/trade/business; and (5) principal working status as domestic duties/unemployed/other/in school/too young or too old and no subsidiary work reported. For age categories 1-3 and 7, we do not assign industry categories and group the members by age-sex only.

24 This approach has been recently used by Geruso (2012) to estimate the fraction of the US mortality gap between black and white people that is due to socio-economic status and by Spears (2013) to estimate the fraction of the gap in average child height between India and sub-Saharan Africa than can be accounted for by exposure to open defecation.

## Appendix A. Additional Tables and Figure

**Table A1:** Districts with Larger Declines in IMR Saw Bigger Decreases in Calorie Consumption - District- level Analysis (National Sample Survey, 1987/88 to 2004/05)

Outcome (calorie type):	Cereals (1)	Cereals (2)	All (3)	All (4)
Infant mortality rate	1.205**	1.002	1.953**	0.927
	(0.608)	(0.741)	(0.836)	(0.904)
Round 61 (2004/05)	-181.433***	-273.311***	-80.970***	-255.631***
	(22.255)	(43.999)	(28.828)	(51.903)
Log of real MPCE, in Rs 1987/88		294.443***		742.571***
		(58.177)		(63.260)
Urban		-312.378*		-256.024
		(183.257)		(214.767)
F-statistic: IMR = 1	0.114	0.000	1.301	0.006
p-value	0.736	0.998	0.255	0.936
*N* (districts)	390	390	390	390
*N* (district-years, i.e. observations)	780	780	780	780

*Notes.* Heteroscedasticity-robust standard errors are shown in parentheses. Sample consists of districts; districts in the bottom and top 2% of calorie distribution were excluded from the sample. All regressions include district fixed effects. MPCE stands for ‘monthly *per capita* expenditures’, which is adjusted for inflation using the CPIAL for rural households and CPIIW for urban households. Controls for share of district population that is Muslim, non-Hindu & non-Muslim, scheduled caste, scheduled tribe and primary household occupation were added to regression models in columns (2) and (4). ***p < 0.01, **p < 0.05, *p < 0.10.

**Table A2:** Infant Mortality and Calorie Consumption: Robustness Checks with Extended Demographic Controls

	Corresponding column in Table 3		
	(5)	(6)	(7)
Panel (*a1*): Cereal calories, without extended dempographic controls Percentage of households without toilet in PSU	1.486***(0.272)	0.580**(0.259)	1.310***(0.309)
Panel (*a2*): Cereal calories, with extended dempographic controls Percentage of households without toilet in PSU	1.476***-0.27	0.619*(0.257)	1.340***(0.304)
Panel (*b1*): Total calories, without extended dempographic controls Percentage of households without toilet in PSU	1.060***(0.231)	0.565**(0.227)	0.438*(0.260)
Panel (*b2*): Total calories, with extended dempographic controls Percentage of households without toilet in PSU	1.056***(0.230)	0.605**(0.225)	0.473+(0.256)
Main controls	X	X	X
Social groups	Education	X		
Cereal prices		X	
Local average MPCE			X

*Notes.* Panels (*a1* and *b1*) correspond exactly with Table 3, and are repeated for comparison. Panels (*a2*) and (*b2*) add the extended demographic controls of Table 4: indicators for the number of married males and females and of teenagers.
