# Supplementary material for: Health and Hunger: Disease, Energy Needs, and the Indian Calorie Consumption Puzzle*
Source: Econ J (London). 2017 Apr 27;127(606):2378–409. doi: 10.1111/ecoj.12417 (PMC7797625; doi:10.1111/ecoj.12417)
Supplement: Supplementary file 1 [file TEJ-127-606-2378-s001.zip › data for replication/IHDS results/README.docx]

**ihds od – revision 2016**

This is a Stata do file. It produces the tables which study the link between open defecation and calorie consumption, using the India Human Development Survey. It starts with the publicly available IHDS file 22626-0002-Data.dta, which is available for free from the IHDS team at ihds.umd.edu.

This do file converts survey consumption data into calories using the Gopalan conversion factors that are used by Deaton and Dreze. It computes PSU average od, saves it, and merges it at the PSU level. It produces Table 3, Table 5, and Appendix Table 2.

**ihds imr**

This do file starts where the “ihds od” do file leaves off, and produces Table 4. It does this by merging in collapsed PSU level IMR from the birth recode, which is included with these replication files as ihdsimr.dta. Note that both of these do files “trim” from the regression the top and bottom 1% of the calorie data.

**ihds BMI**

This do file starts with the same leave off, and produces Table 6, the regression of adult women’s BMI on open defecation and controls. Because the main do file uses the *household* recode, this do file merges in two bits of data from the *individual* recode: BMI.dta and ihdsheight.dta. These are included with this replication folder.
